# A Bio-Inspired Compliance Planning and Implementation Method for Hydraulically Actuated Quadruped Robots with Consideration of Ground Stiffness

**DOI:** 10.3390/s21082838

**Published:** 2021-04-17

**Authors:** Xiaoxing Zhang, Haoyuan Yi, Junjun Liu, Qi Li, Xin Luo

**Affiliations:** State Key Laboratory of Digital Manufacturing Equipment and Technology, Huazhong University of Science and Technology, Wuhan 430074, China; xiaoxingzhang@hust.edu.cn (X.Z.); yihaoyuan@hust.edu.cn (H.Y.); liujunjun@hust.edu.cn (J.L.); qili@hust.edu.cn (Q.L.)

**Keywords:** active compliance control, stiffness control, compliance planning, quadruped robots, harmonic locomotion

## Abstract

There has been a rising interest in compliant legged locomotion to improve the adaptability and energy efficiency of robots. However, few approaches can be generalized to soft ground due to the lack of consideration of the ground surface. When a robot locomotes on soft ground, the elastic robot legs and compressible ground surface are connected in series. The combined compliance of the leg and surface determines the natural dynamics of the whole system and affects the stability and efficiency of the robot. This paper proposes a bio-inspired leg compliance planning and implementation method with consideration of the ground surface. The ground stiffness is estimated based on analysis of ground reaction forces in the frequency domain, and the leg compliance is actively regulated during locomotion, adapting them to achieve harmonic oscillation. The leg compliance is planned on the condition of resonant movement which agrees with natural dynamics and facilitates rhythmicity and efficiency. The proposed method has been implemented on a hydraulic quadruped robot. The simulations and experimental results verified the effectiveness of our method.

## 1. Introduction

Legged robots have superior mobility and maneuverability in complex unstructured environments, benefitting from the ability afforded by their morphology and varied gaits [1]. Recent years have witnessed significant achievements in the research area of legged robots. Versatile high-performance robots, such as BigDog, Spot, and Atlas developed by Boston Dynamics [2], the MIT cheetah series [3,4,5,6,7], the HyQ [8,9] and the ANYmal [10,11] developed by IIT and ETH Zurich, the Aliengo [12] from the Unitree Robotics, and the Jueying robots [13,14] developed by the DeepRobotics and Zhejiang University, have brought prospective practical applications. Nevertheless, despite considerable performance improvements in the past 20 years in mechatronics and control, the locomotion efficiency of the state-of-the-art robots lags far behind that of their biological counterparts [15]. The evolutionary process of thousands of years has endowed legged animals with an exquisite dynamic mechanism and achieved excellent motion performance. Learning the dynamic mechanism from legged animals is the inevitable way to further improve the performance of legged robots.

An essential property of animal locomotion is the alternative foot-ground contact in the swing and support phases of a locomotor cycle based on their inherent dynamics, roughly defining the locomotion’s rhythmicity [16,17]. Elastic structures and spring-like leg behavior have been widely found in the locomotion of animals, and the spring-loaded inverted pendulum (SLIP) model has been abstracted into a template to resolve the redundancy of multiple legs and joints [18,19]. The inherent parameters of the dynamic system, namely the body mass and the compliance of the leg, determine the rhythmicity of legged locomotion and influence the stability and efficiency. Elastic structures can be appropriately arranged to minimize metabolic energy costs in movements that are oscillatory naturally on the condition that the dynamic parameters match the locomotion rhythmicity [20].

An emerging amount of research in biomechanics and kinesiology has revealed that elastic structures, including tendons, ligaments, muscles, and foot pads [21], play an important role in rhythmicity modulation. Legged animals actively change their spring leg stiffness to adapt to terrain, speed, gait and loads, resulting in stable and efficient resonant locomotion. Ferris and Farley found that, within a certain range of ground stiffness, human leg stiffness increased significantly on soft ground to ensure the stiffness of the series leg–surface combination remained constant and that the entire adjustment process could be completed in one gait cycle [22,23]. Kim and Park observed that human leg stiffness increased with speed to modulate step frequency, indicating that the movement of the center of mass (CoM) in the stance phase may take advantage of harmonic oscillations of the compliant leg [24]. Silder and Besier et al. estimated the dimensionless leg stiffness of running humans with an added load and revealed that subjects run with higher leg stiffness to accommodate the load [25]. Cavagna and Legramandi studied animals of different sizes and running humans at different ages locomoting with varied gaits and found that the step frequency roughly equals the resonant frequency in trotting and running, whereas it was about half the resonant frequency in hopping [26]. The analysis of locomotion data suggests that legged animals exploit elastic properties of muscles, tendons and skeletal elements to adjust leg compliance [27,28], and the vertical oscillations of the stance leg are aligned with the step frequency [29]. Humans, and by generalization all animals, keep resonance during locomotion [26,30].

To approach the performance and efficiency of the biological archetype, researchers have expended plenty of endeavors in realizing legged locomotion in theory and engineering practice and made numerous profound achievements [18,31]. Raibert has made seminal contributions to dynamic legged locomotion, realizing trotting, pacing, and bounding gaits by quadruped robots using the three-part locomotion algorithms [32,33,34]. However, the high energy consumption limits the further application of this approach to a certain extent. The MIT Biomimetic Robotics Lab led by S. Kim has demonstrated highly dynamic trotting, trot-running, bounding, pronking gaits, and backflips on the quadruped robot family of Cheetahs [3,4,6,35,36,37,38,39,40,41]. Through the impulse scaling of foot reaction forces obtained from biological research, the Cheetah robots achieved high performance in terms of locomotion efficiency and agility without much concern regarding contact dynamics. The Dynamic Legged Systems lab in IIT, Italy, investigated the active impedance control methods for hydraulic robots and demonstrated the versatility and terrain adaptability of the hydraulic quadruped robot HyQ [8,9,42]. To overcome the limitations of the presence of unmodeled contact dynamics, they recently proposed a novel soft terrain estimation and adaptation algorithm to maintain consistent compliant contact which was partly based on state estimation to calculate the penetration of the feet [43,44]. Nevertheless, it may be difficult to extend this to other robots due to the requirements of a variety of sensors with high accuracy, such as a tactical-grade Inertial Measurement Unit (IMU).

To achieve tunable compliance of the robot leg, it is crucial to employ physical or virtual elasticity in drive units. Physical elasticity means that the drive unit consists of some physical elastic components, and may be actively controlled to adjust the stiffness. Virtual elasticity means that the elasticity is achieved by an active compliance control algorithm. One approach to the implementation of physical elasticity is to add elastic components to the leg [45,46,47], and another method is to mount them between the actuator and robots to form a serial elastic actuator (SEA) [10,48,49] or variable stiffness actuator (VSA) [50,51]. However, these methods make robot structure design and motion control more difficult, as well as restricts its application due to the limited range of stiffness. Comparatively, the virtual elasticity methods, through active compliance control, such as the impedance [8,9,52] or admittance control [53,54], the virtual model control (VMC) [55], and the vertical impulse scaling of ground reaction force [35,38,39] demonstrate better adaptability. These methods improve the robot’s performance in challenging terrains due to the wider range of stiffness adjustment capability. Unfortunately, the performance of active control methods is still subject to the responsiveness of the actuator to a great extent in practical applications.

This paper proposes a systematic compliance planning and implementation method for a quadrupedal robot on various terrains. The stiffness of the ground surface is estimated during locomotion based on the analysis of ground reaction forces in the frequency domain. The compliance of the robot leg is actively controlled to offer virtual elasticity and is regulated as changes of locomotion parameters and the environment to achieve harmonic oscillation of the elastic leg-ground system in the stance phase.

In developing the bio-inspired compliance planning and implementation method, we mainly offer two contributions: (a) A novel surface stiffness estimation method is proposed for legged robots. Through analysis of ground reaction forces in the frequency domain, the estimation can be completed within one step period. (b) The principle of harmonic locomotion is exploited for the leg compliance planning to improve rhythmicity and efficiency. The leg compliance is actively regulated on the condition of resonant movement which agrees with the natural dynamics of the leg-ground system.

The paper is organized into six sections: Section 2 describes the surface stiffness estimation and the compliance profile planning method. Section 3 presents the framework for the resonant locomotion of a quadruped robot. Section 4 illustrates the implementation of the proposed method on a hydraulically actuated quadruped robot. Section 5 presents the simulation and practical experiments. Finally, conclusions are drawn in Section 6.

## 2. Compliance Planning for Harmonic Locomotion

As stated before, legged animals exploit elastic properties and adjust leg compliance to maintain longitudinal harmonic oscillation. Inspired by biological research, this paper takes harmonic locomotion as a basic principle for the motion planning and control of a quadruped robot. The leg compliance is planned on the condition of resonance to exploit the natural dynamics of elastic leg and to match the desired motion in terms of locomotion rhythmicity on various ground surfaces.

### 2.1. Principles of Harmonic Locomotion

**Figure 1:**
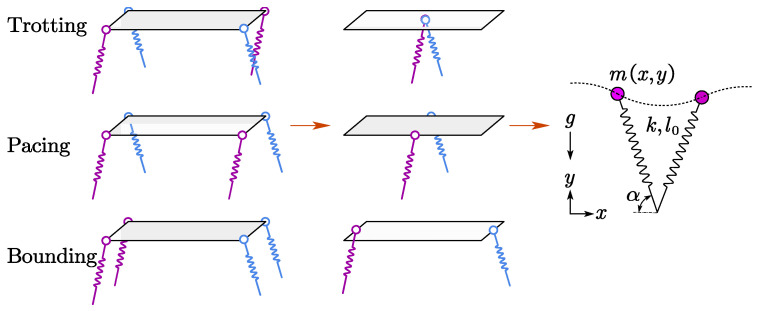
The SLIP model for quadruped robots. The four legs of a robot moving at trotting, pacing, or bounding gait can be simplified as a single equivalent virtual leg by seeking synergies and symmetries. The stiffness of virtual leg *k* in SLIP model roughly equals that of the quadruped robot, and the point mass *m* at the CoM is about half the body mass of the robot moving at a symmetrical gait.

The dynamics of legged locomotion can be revealed by the SLIP model, and various locomotion gaits of quadruped robots such as trotting, pacing, and bounding are expressed as elastic oscillations of the sample mass-spring bouncing system in SLIP, as illustrated in Figure 1. Ideally, harmonic motion at resonance can be realized on the mass-spring bouncing system based on natural passive dynamics without any energy consumption.

However, the natural dynamics of robots are actively controlled and the motion is arbitrarily generated, respectively; thus, the harmonic motion can hardly be achieved directly. In pursuit of efficient harmonic motion, the dynamics determined by the actively controlled joints should agree with the rhythmicity of motion, and foot reaction forces should match the oscillation of the CoM during each stance phase.

For a simple spring-mass bouncing system, the natural dynamics are governed by

(1) $$f=\frac{1}{2\pi }\sqrt{\frac{k}{m}}$$

where *k* is the stiffness of the spring, *m* is the center mass, and *f* is the natural bouncing frequency. The natural dynamics described by *k* and *m* determine the passive harmonic motion parameterized by *f*.

For quadruped robots locomoting on various ground surfaces, the compressible ground surface and compliant leg are connected in series to form an elastic combination, and the combined effective stiffness regulates the legged motion on ground surfaces. To achieve harmonic locomotion for the quadruped robot, the estimation of the surface compliance under the current robot statuses and the prediction of the leg compliance for the next step based on the estimation are the main issues for the compliance planning.

### 2.2. Surface Stiffness Estimation

**Figure 2:**
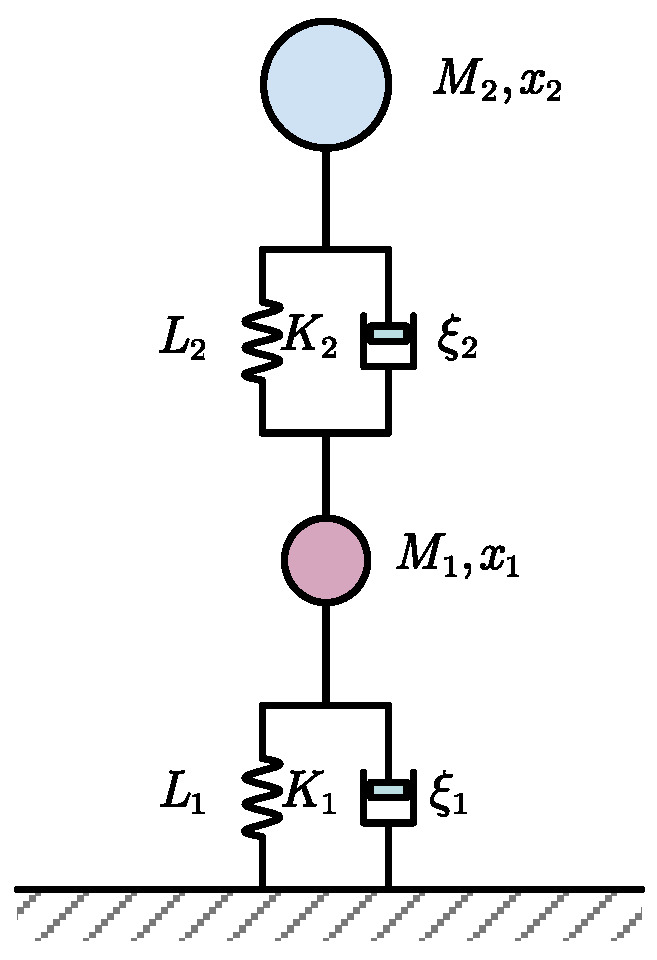
The robot–ground contact model.

In the practical quadruped robot, the contact force can be directly and precisely measured while the deformation value estimated from the robot state is usually not reliable. Thus, the surface stiffness estimation should mainly be based on the foot reaction forces. The robotground contact model is shown in Figure 2, where *M*_1_ and *M*_2_ represent the mass of the foot and body mass of the robot and, $K_{1},\xi_{1}$, $K_{2},\xi_{2}$ denote the stiffness and damping of the surface and the compliant leg, respectively.

The simplified robot–ground model possesses two degrees of freedom. Selecting the height of the foot *x*_1_ and the height of the robot mass *x*_2_ as the generalized coordinates, the Lagrangian Equation for the system is given by

(2) $$\frac{d}{d(t)}\left(\frac{\partial T}{\partial \dot{q}}\right)-\frac{\partial T}{\partial q}+\frac{\partial V}{\partial q}+\frac{\partial D}{\partial \dot{q}}=Q$$

where *T*, *V*, *D* and *Q* denote the kinetic energy, potential energy, dissipated energy, and general forces of the system, respectively, and the details are given as

(3) $$\{\begin{array}{l} T=\frac{1}{2}\left(M_{1}{\dot{x}}_{1}^{2}+M_{2}{\dot{x}}_{2}^{2}\right)\\ V=M_{2}g\left(L_{1}+L_{2}-x_{2}\right)+M_{1}g\left(L_{1}-x_{1}\right)+\frac{1}{2}K_{2}{\left(x_{1}-x_{2}\right)}^{2}+\frac{1}{2}K_{1}x_{1}^{2}\\ D=\frac{1}{2}\xi_{2}{\left({\dot{x}}_{1}-{\dot{x}}_{2}\right)}^{2}+\frac{1}{2}\xi_{1}{\dot{x}}_{1}^{2}\\ Q=0 \end{array}$$

Assuming that the damping of the spring leg and ground surface is negligible, the dynamics Equation (2) can be converted into

(4) $$\left[\begin{array}{c} M_{1}\\ M_{2} \end{array}\right]\left[\begin{array}{c} {\ddot{x}}_{1}\\ {\ddot{x}}_{2} \end{array}\right]+\left[\begin{array}{cc} K_{1}+K_{2} & -K_{2}\\ -K_{2} & K_{2} \end{array}\right]\left[\begin{array}{c} x_{1}\\ x_{2} \end{array}\right]=\left[\begin{array}{c} M_{1}g\\ M_{2}g \end{array}\right]$$

To solve the Equation (4), the variable transformation

(5) $$a=\frac{K_{1}+K_{2}}{M_{1}},b=\frac{K_{2}}{M_{1}},c=\frac{K_{2}}{M_{2}},d=\frac{K_{2}}{M_{2}}$$

is used to obtain a relatively simple form, as follows:

(6) $$\{\begin{array}{l} {\ddot{x}}_{1}+ax_{1}-bx_{2}=0\\ {\ddot{x}}_{2}-cx_{1}+dx_{2}=0 \end{array}$$

Considering the analytical solutions of the ordinary differential Equation system (6) have the same frequency and different amplitudes, then the solutions can be assumed to be

(7) $$\{\begin{array}{l} x_{1}=u_{1}f(t)\\ x_{2}=u_{2}f(t) \end{array}$$

and Equation system (6) can be transformed into

(8) $$\ddot{f}(t)+\lambda f(t)=0$$

The solutions for Equation (8) generally have the following form

(9) f(t)=Ccos(ωt−φ)

Equation (9) can be substituted into (6) to generate

(10) $$\{\begin{array}{l} \left(a-\omega^{2}\right)u_{1}-bu_{2}=0\\ -cu_{1}+\left(d-\omega^{2}\right)u_{2}=0 \end{array}$$

To ensure the existence of the solution, the determinant of Equation (10) should be zero, that is

(11) $$\left|\begin{array}{cc} a-\omega^{2} & -b\\ -c & d-\omega^{2} \end{array}\right|=0$$

Equation (11) can be converted into an algebraic equation

(12) $$\omega^{4}-(a+d)\omega^{2}+(ad-bc)=0$$

The analytical solutions of Equation (12) can be derived as

(13) $$\omega_{1,2}^{2}=\frac{1}{2}(a+d)\pm \frac{1}{2}\sqrt{{\left(a-d\right)}^{2}+4bc}$$

where

$$a=\frac{K_{1}+K_{2}}{M_{1}},b=\frac{K_{2}}{M_{1}},c=\frac{K_{2}}{M_{2}},d=\frac{K_{2}}{M_{2}},\omega_{1,2}=2\pi f_{1,2}$$

Thus, the two modes of vibration for the robot-ground system are fully developed and can be expressed by Equations (5) and (13). On the assumption of the negligible damping of the ground surface, the foot reaction force is directly proportional to the displacement. Therefore, the vibration mode of the foot reaction force and that of the displacement, and the current spring leg stiffness and surface stiffness can be calculated by solving the vibration mode of the foot reaction force with Equation (13).

### 2.3. Leg Compliance Planning

According to the SLIP model of legged locomotion, the whole gait cycle period *T* consists of the aerial phase period *T*_a_ and stance phase period *T*_s_; that is

(14) $$T=T_{a}+T_{s}$$

The stance phase period can also be expressed as $T_{s}=\gamma T$, where γ denotes the duty factor.

The longitudinal oscillations of the stance leg during the stance phase approximately operate as a part of the simple harmonic vibration. The coefficient *c* is used to relate the stance phase period *T*_s_ and harmonic vibration period *T*_h_, and we can thus obtain

(15) $$T_{h}=cT_{s}=c\gamma T$$

The relation between vibration frequency *f*_s_ and gait step frequency *f*_step_ can be expressed as

(16) $$f_{s}=f_{step}/c\gamma$$

The synthetical stiffness *K* of the combined robot–ground system determines the resonant frequency *f*_s_:

(17) $$2\pi f_{s}=\sqrt{\frac{K}{m}}$$

The preferred synthetical stiffness *K* can be calculated from Equations (16) and (17):

(18) $$K=4m\pi^{2}f_{s}{}^{2}=4m\pi^{2}f_{step}{}^{2}/c^{2}\gamma^{2}$$

Through the relationship between the synthetical stiffness *K* and surface stiffness *K*_1_, leg stiffness *K*_2_ is governed by

(19) $$K=\frac{K_{surf}K_{leg}}{K_{surf}+K_{leg}}$$

The preferred leg stiffness can be derived from Equations (18) and (19) and can be expressed by

(20) $$K_{leg}=\frac{KK_{surf}}{K_{surf}-K}=\frac{4Km\pi^{2}f_{\mathrm{step}\;}^{2}/c^{2}\gamma^{2}}{K_{surf}-4m\pi^{2}f_{\mathrm{step}\;}^{2}/c^{2}\gamma^{2}}$$

Equation (20) can be used to estimate the preferred leg stiffness *K_leg_* based on the gait characteristics of legged locomotion.

## 3. Control Framework for Harmonic Locomotion of a Quadruped Robot

**Figure 3:**
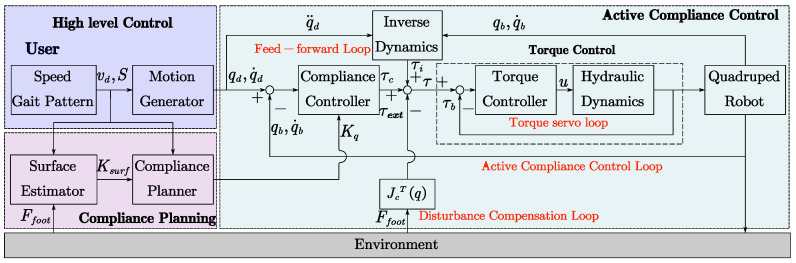
Block diagram of the bio-inspired compliance planning and control framework for the quadruped robot.

The implementation of the active compliance control and planning for the quadruped robot is depicted in Figure 3. The entire framework consists of high-level control, compliance planning, and active compliance control with the inner torque control. The high-level control is the interaction interface between users and the robot. Users set the moving speed and gait pattern for the robot motion generator with the high-level controller and then the desired motion profile is produced. The compliance planning firstly estimates the compliance of the ground surface based on the reaction forces of robot feet and the current motion states. Then, the compliance planner calculates the preferred leg compliance, as is presented in Section 2. The active compliance control includes an inner torque control loop and an outer compliance control loop. Besides, a feed-forward loop based on rigid-body inverse dynamics is used to enhance the accuracy of motion control, and a disturbance compensation loop is introduced to deal with external forces such as loads. It should be noted that the motion generator is mainly based on the SLIP model and is not discussed in detail in this paper, and the minimum angle-of-attack is set as 62° [56] to guarantee the assumption of non-slip contact limited by the friction cone.

## 4. Implementation for a Hydraulically Actuated Quadruped Robot

This section presents our implementation of the bio-inspired compliance planning for a hydraulically actuated quadruped robot. In contrast to electric motors, the main superiority of hydraulic actuation is its high power density, which is critical for heavy-duty legged robots. On the other hand, the control of hydraulic actuation is more challenging because of the wide variety of nonlinearities in the system. This section presents the detailed implementation of the compliance planning methods for the hydraulically actuated quadruped robot based on the framework in Section 3.

### 4.1. Overview of the Hydraulically Actuated Quadruped Robot

**Figure 4:**
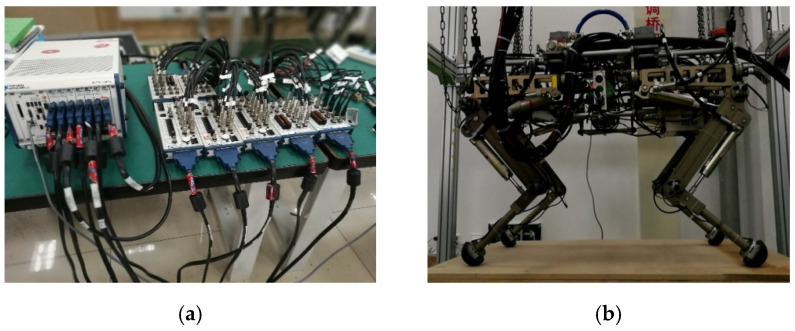
Overview of the hydraulically actuated quadruped robot system. (**a**) National Instrument^®^-based controller; (**b**) prototype of the hydraulically actuated quadruped robot.

Figure 4 shows the overview of the hydraulically actuated quadruped robot system. The prototype of the quadruped robot is hydraulically powered by an off-board pump. It features three active degrees of freedom (DOF) per leg: two flexion/extension DOF in the hip and knee joint, and an abduction/adduction DOF for lateral swing. Each joint is actuated by a hydraulic cylinder (Linear cylinder LB, Hoerbiger) controlled by a servo valve (G761, Moog). The geometric dimensions of the robot are 1000 × 660 × 800 mm (length/width/height) and the total mass is about 75 kg. A variety of sensors are equipped on the robot including a 16-bit high-precision digital encoder (SROA35, Reagle) at each DOF, two pressure sensors (511-943, Huba Control) for each hydraulic cylinder, a three-axis force sensor (S302, SRI) for ground reaction force sensing at each foot, and a high-performance IMU (MTi-30, Xsens) for the state estimation of the robot torso. It is controlled by a National Instrument^®^ based controller.

### 4.2. Force Control of the Hydraulic Actuator

**Figure 5:**
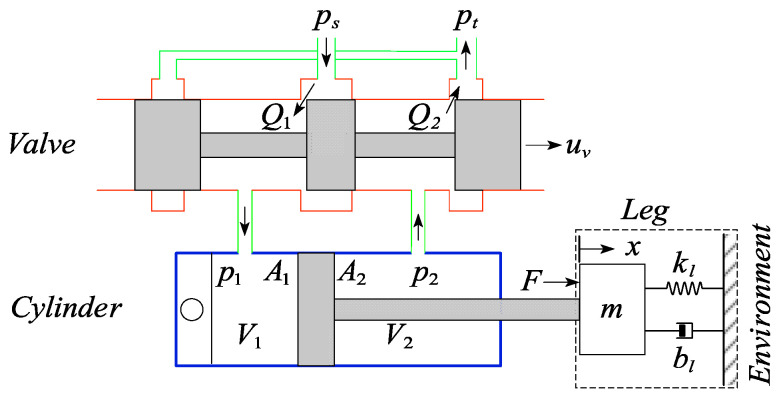
Schematic diagram of the single-rod hydraulic system.

The hydraulic actuator of the quadruped robot under consideration is depicted in Figure 5. As is shown, a servo valve controlled single-rod hydraulic cylinder exerts pressure force on the robot leg link. Nonlinearity in the system, such as the compressibility of the hydraulic fluid and the flexibility of the tubing, the complex flow characters of the servo valve, and the nonlinear dynamic friction of the hydraulic cylinders, significantly affects the performance of the system. The nonlinear dynamics of the hydraulic system are modeled in this paper to improve the control performance.

The pressure dynamics of the cylinder considering the compressibility of the oil can be modeled as

(21) $$\begin{array}{l} \frac{V_{1}}{\beta }{\dot{p}}_{1}=Q_{1}-A_{1}\dot{x}-c_{i}(p_{1}-p_{2})\\ \frac{V_{2}}{\beta }{\dot{p}}_{2}=A_{2}\dot{x}-Q_{2}+c_{i}(p_{1}-p_{2}) \end{array}$$

where $V_{1}=V_{01}+A_{1}x$ and $V_{2}=V_{02}-A_{2}x$ denote the total control volumes of the cylinder chambers, $V_{01}$ and $V_{02}$ are the original volumes when x=0,x is the piston displacement, β is the oil bulk modulus,$Q_{1}$ is the supplied flow rate,$Q_{2}$ is the return flow rate and $c_{i}$ is the internal leakage flow coefficient.

The considered servo valve is developed for high dynamic response applications. The dynamics of the servo valve are neglected; thus the control input u is assumed to be proportional to the valve spool displacement x. $Q_{1}$ and $Q_{2}$ are related to the control input u by

(22) $$\begin{array}{l} Q_{1}=s(u)c_{1}u\sqrt{p_{s}-p_{1}}+s(-u)c_{2}u\sqrt{p_{1}-p_{r}}\\ Q_{2}=s(u)c_{3}u\sqrt{p_{2}-p_{r}}+s(-u)c_{4}u\sqrt{p_{s}-p_{2}} \end{array}$$

where $c_{1},c_{2},c_{3},c_{4}$ are the valve orifice coefficients, $p_{s}$ is the pressure of fluid supply and $p_{r}$ is the reservoir or reference pressure. The function s(u) is defined as

(23) $$s(u)=\{\begin{array}{ll} 1, & \;\mathrm{if}\;u\geq 0\\ 0, & \;\mathrm{if}\;u<0 \end{array}$$

The net fluid force can be described by

(24) $$F=p_{1}A_{1}-p_{2}A_{2}$$

where $p_{1}$ and $p_{2}$ are the pressures inside the two chambers of the cylinder and $A_{1}$ and $A_{2}$ represent the piston area and piston rod area, respectively. The effective force applied on the loads is given by

(25) $$f=F-\tilde{f}(t)$$

where $\tilde{f}(t)$ is the estimation of the friction force.

The time derivative of Equation (24) is given by

(26) $$\dot{F}={\dot{p}}_{1}A_{1}-{\dot{p}}_{2}A_{2}$$

Substituting Equations (21) and (22) into (26) yields

(27) $$\dot{F}=-\dot{x}\beta (\frac{A_{1}{}^{2}}{V_{1}}+\frac{A_{2}{}^{2}}{V_{2}})-c_{i}(p_{1}-p_{2})\beta (\frac{A_{1}}{V_{1}}+\frac{A_{2}}{V_{2}})+z(x,p_{1},p_{2})u$$

where

(28) $$z(x,p_{1},p_{2})=\frac{\beta }{V_{1}}(s(u)c_{1}\sqrt{p_{s}-p_{1}}+s(-u)c_{2}\sqrt{p_{1}-p_{r}}+s(u)c_{3}\sqrt{p_{2}-p_{r}}+s(-u)c_{4}\sqrt{p_{s}-p_{2}})$$

Equation (27) maps the control voltage to the fluid force. Through the inverse of Equation (27), the hydraulic force controller can be obtained as

(29) $$u=\frac{1}{z}({\dot{F}}_{d}-k_{L}(F-F_{d})+\dot{x}\beta (\frac{A_{2}{}^{2}}{V_{2}}+\frac{A_{1}{}^{2}}{V_{1}}))$$

where $F_{d}$ denotes the desired force of the hydraulic cylinder,$k_{L}$ is a positive gain coefficient of force error, and the non-zero item *z* is presented in Equation (28). We see that $\dot{F}$ in Equation (27) becomes

(30) $$\dot{F}={\dot{F}}_{d}-k_{L}(F-F_{d})$$

The exponential force stabilization is guaranteed by

(31) $$\left(F-F_{d})={\hat{e}}^{k_{L}t}(F(0)-F_{d}(0)\right)$$

Equation (31) indicates that $(F(t)-F_{d}(t))\to 0$ with time constant $\tau =1/k_{L}$. The value of the time constant mainly depends on the response bandwidth of the hydraulic system. The model-based force controller governed by Equation (29) captures the nonlinear dynamics of the hydraulic actuation system and achieves compensation through feedback linearization.

### 4.3. Active Compliance Controller Design

Active compliance control plays an important role in the period of contact of the actuator and the load. It indicates the synchronous control of force and position during the contact by tuning the stiffness, damping and inertia, and can be described as

(32) $$f_{c}=K_{p}(x_{ref}-x)+K_{d}({\dot{x}}_{ref}-\dot{x})+K_{m}({\ddot{x}}_{ref}-\ddot{x})$$

where $f_{c}$ is the contact force and $K_{p}$, $K_{d}$ and $K_{m}$ indicate the stiffness, damping, and inertia parameters, respectively; $x_{ref}$ denotes the desired position reference and x is the measured position.

Referring to Equation (32), position and velocity tracking errors are used to compute the desired force. The measured acceleration is usually unreliable since it is calculated through the second-order difference of the position signals. The desired acceleration is used to replace the acceleration feedback. Thus, the ideal desired force is derived as

(33) $$f_{d}=m{\ddot{x}}_{d}(t)+k_{v}({\dot{x}}_{d}(t)-\dot{x}(t))+k_{p}(x_{d}(t)-x(t))$$

where m, $k_{v}$, $k_{p}$ are the equivalent mass of the load, the velocity feedback gain and the position feedback gain, respectively; $x_{d}(t)$ and x(t) represent the desired and measured positions of the piston.

In practice, the friction force of the hydraulic cylinder also affects the character of the contact. In hydraulic systems, notable friction force exists in hydraulic cylinders for leak tightness requirements. A dynamic friction force identification method is used based on our previous work [49]. For further research on dynamic friction identification methods, one can refer to some state-of-the-art works [57,58]. Therefore, in this paper the desired fluid force is

(34) $$F_{d}=m{\ddot{x}}_{d}(t)+k_{v}({\dot{x}}_{d}(t)-\dot{x}(t))+k_{p}(x_{d}(t)-x(t))+{\hat{F}}_{f}(\dot{x})+{\hat{F}}_{ext}$$

where ${\hat{F}}_{f}$ is the estimated friction force, and ${\hat{F}}_{ext}$ is the estimated external disturbance. The disturbance item is set as a constant of the robot gravity in the stance phase and is reduced to zero during the swing phase. The equation of motion is given by

(35) $$F-F_{f}(\dot{x})-F_{ext}=m\ddot{x}(t)$$

where $F_{f}(\dot{x})$ and $F_{ext}$ are the friction force and external disturbances, respectively. Subtracting (34) from (35) and letting $e=x-x_{d}$, we get

(36) $$m\ddot{e}+k_{v}\dot{e}+k_{p}e=(F-F_{d})+\delta (\dot{x})$$

where $\delta (\dot{x})={\hat{F}}_{f}(\dot{x})+{\hat{F}}_{ext}-F_{f}(\dot{x})-F_{ext}$ denotes the disturbance due to inaccurate estimation of friction and external disturbances. The Equation (36) can be considered a second-order linear system in e driven by $\left(F-F_{d}\right)$ and $\delta (\dot{x})$. The disturbance $\delta (\dot{x})$ is bounded and $(F(t)-F_{d}(t))\to 0$, so the system represented by the Equation (36) is stable.

Equations (29) and (34) reveal the main framework of the active compliance controller for the hydraulic quadruped robot. The velocity gain $k_{v}$ is equivalent to damping, and the position gain $k_{p}$ serves as the spring stiffness. The damping $k_{v}$ is set as a constant, and the active stiffness is regulated online to achieve the desired compliance of the robot.

It should be noted that the active compliance parameters in the Equation (34) are expressed in the actuation space. Based on the virtual work principle, the relation between the stiffness in each actuation and joint space can be derived as

(37) $$k_{\theta }=J_{c}{}^{T}k_{l}J_{c}=l_{c}{}^{2}k_{l}$$

where $k_{\theta }$ is the rotational stiffness in joint space,$k_{l}$ is the linear stiffness in cylinder actuation space and $l_{c}$ denotes the force arm of cylinder.

As is known, the Jacobian relates the joint torques and the forces applied on the foot by

(38) $$\tau =J^{T}F$$

From the definition of stiffness, we differentiate Equation (38) and we have

(39) $$\underset{K_{\theta }}{\underbrace{\frac{\partial \tau }{\partial \theta }}}=\frac{\partial (J^{T}F)}{\partial \theta }=(\frac{\partial J^{T}}{\partial \theta })F+J^{T}\underset{K_{X}}{\underbrace{\frac{\partial F}{\partial X}}}\underset{J}{\underbrace{\frac{\partial X}{\partial \theta }}}$$

where J is the Jacobin of the leg and θ is the joint angle; $K_{\theta }$ and $K_{X}$ represent the stiffness matrixes in the joint space and the Cartesian space. We approximate the vertical stiffness in $K_{X}$ by taking the value of the leg stiffness obtained from Equation (20), and the forward and lateral stiffnesses are set as a reasonable constant based on experience. Equation (37) and (39) map the vertical leg stiffness obtained from Equation (20) into that in the cylinder actuation space.

## 5. Experiments and Simulations

The systematic method of compliance planning and implementation proposed in this paper consists of two major steps: the compliance planning and the compliance implementation. For the compliance implementation, the position and force tracking experiments were conducted to verify the inner force controller and the outer position controller of the active compliance controller, as illustrated in Section 5.1; to verify the superiority over the traditional PID-based position controller, a comparative impact disturbance experiment was conducted in Section 5.2. For the compliance planning, the ground surface compliance estimation experiment was conducted in Section 5.3 to demonstrate the reliability of the estimated ground stiffness. Based on the estimated ground stiffness, the compliance planning of the robot leg was realized using the Equation (20). The effectiveness of the whole method was verified through simulation and comparison of energy efficiency in Section 5.4.

### 5.1. Experiments on Position/Force Tracking

In the control framework of this paper, the inner-loop torque control is the basis of the compliance control algorithm, while, ideally, the compliance control will not reduce the position tracking performance. To verify the force and position tracking performance of the compliance controller, the experiment was conducted on the left front leg of the quadruped robot hanging in the air. The robot was controlled to perform a 50 mm range of squatting motion when a 25 kg load was mounted on the foot. The pressure sensors on the hydraulic cylinder allow for the calculation of the measured force of the hydraulic cylinder. The encoder on the joint allows for the indirect measurement of the joint position, which is represented by the cylinder length.

**Figure 6:**
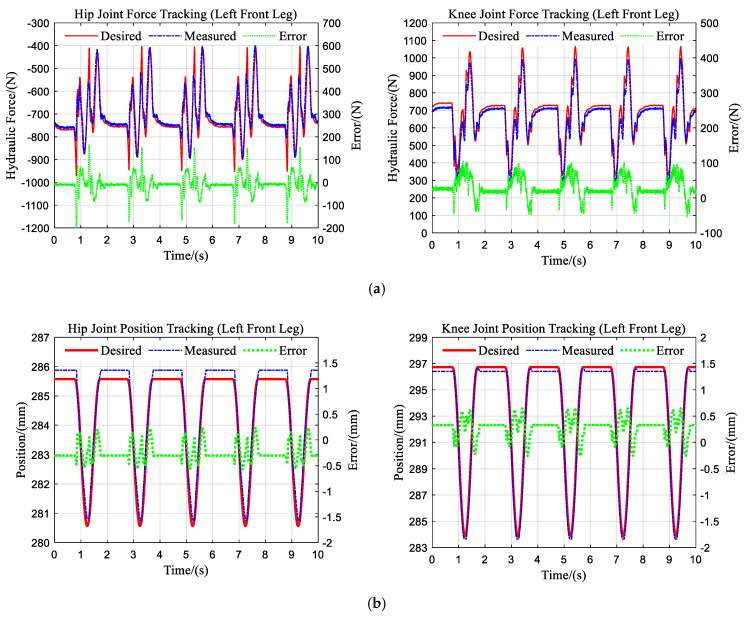
Force/position tracking performance of the active compliance controller on the left front leg. (**a**) Force tracking; (**b**) position tracking.

Figure 6 displays the main results. The force tracking performance of the hip and knee joints is depicted in Figure 6a. As can be seen from the figure, the maximum amplitude of the force tracking errors in the two joints is approximately 100 N. The peak error occurs primarily during the movement of the legs, which may be caused mainly by the disturbance due to the inertial force of the load at the foot. The position tracking performance is provided in Figure 6b, and the maximum amplitude of the errors is about 0.5 mm. The results suggest the promising performance of the active compliance controller and accurate force and position control is achieved at the same time.

### 5.2. Experiments on Impact Disturbance

**Figure 7:**
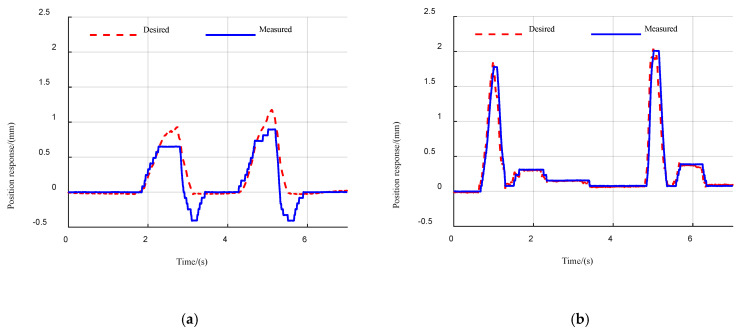
Comparison of desired/measured position response under impact disturbances. (**a**) PID-based position controller; (**b**) proposed controller.

We expect the robot leg to behave as an actual spring under the active compliance controller to cope with the impact disturbances exerted on the robot feet. We designed the corresponding experiment on our quadruped robot. The impact disturbance force was exerted to the foot when the robot was lifted in the air and the position response was measured. Without loss of generality, the actuation stiffness of 500 N/mm was set for the hydraulic cylinder. The impact disturbance force was also sensed by the three-axis force sensor. We took the compression of an ideal virtual spring with the same stiffness as the desired position response and measured the actual position response of the hydraulic cylinder. The comparison is shown in Figure 7. As can be seen from the figure, both the PID-based position controller and the proposed active compliance controller allow the robot to exhibit significant compliance characteristics, similar to the actual spring, given the appropriate gain and proper stiffness. However, there is a significant overshoot in the PID-based position controller, which can seriously affect the stability of the robot. In comparison, the active compliance controller exhibits superior stability.

### 5.3. Experiments on Ground Surface Compliance Estimation

**Figure 8:**
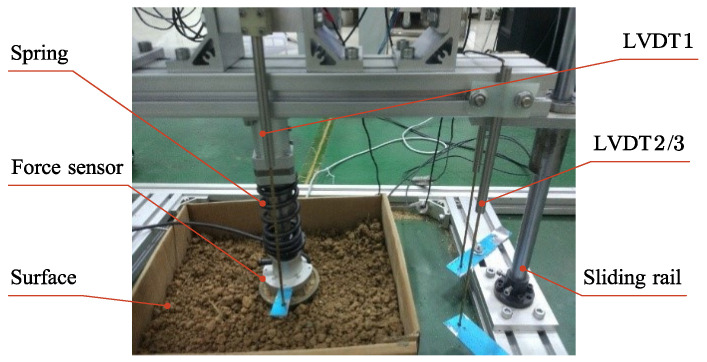
Experimental setup of the single robot leg.

The experimental setup of a single robot leg is built for the study of robot–ground contact behaviors. The internal components, including the spring of the robot leg, substrate surface, sliding rail and sensors are illustrated in Figure 8. The stiffness of the spring was 50 N/mm. The three-axis force sensor was used to measure the contact force. The LVDT (Linear Variable Differential Transformer, GA09, Utsensor) 2/3 were used for the measurement of the displacement of the leg base along the sliding rail; meanwhile, the LVDT 1 was used to measure the deformation of the spring, and the difference between them represented the compression of the ground surface.

**Figure 9:**
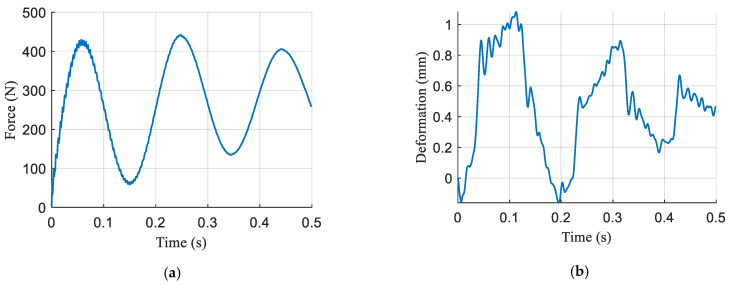
Contact force and surface deformation in the time domain. (**a**) Contact force; (**b**) surface deformation.

The proposed surface compliance estimation method was verified on the experimental setup of a single robot leg. The leg was released after being lifted to a certain height to excite the contact of the robot foot and ground surface. The contact force and surface deformation were measured as shown in Figure 9. Benefitting from various sensing systems in the experimental setup, the stiffness of the ground surface can be calculated by Hooke’s Law. It can be seen in Figure 9 that the maximum contact force is about 440 N, and the maximum deformation is 1.2 mm. The calculation result is 366.7 N/mm, which can be considered as the measured value of surface stiffness.

**Figure 10:**
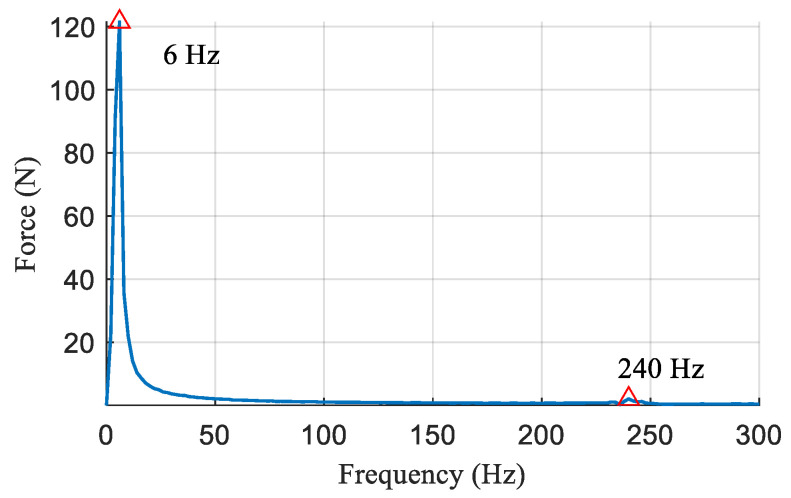
Single-sided amplitude spectrum of contact force.

As can be seen from Figure 9a, a high-frequency oscillation signal appears at the beginning of the period and a low-frequency oscillation signal is present throughout the period. This observation is consistent with the Equation (13). To quantify the frequencies of oscillation signals, the single-sided amplitude spectrum of contact force is obtained by Fast Fourier Transform (FFT) as shown in Figure 10. The result indicates that the contact force mainly contains both a 6 Hz and a 240 Hz component. Substituting the frequencies into the Equation (13), the robot leg stiffness can be obtained as 52.1 N/mm and the ground surface stiffness as 357.2 N/mm. The estimated value of surface stiffness is in general agreement with the measurement. The experiment result verifies the effectiveness of the proposed surface stiffness estimation method in Section 2.2; thus, the ground stiffness can be taken into consideration in the process of compliance planning, as given in Section 2.3.

### 5.4. Simulation and Comparison of Energy Efficiency

**Figure 11:**
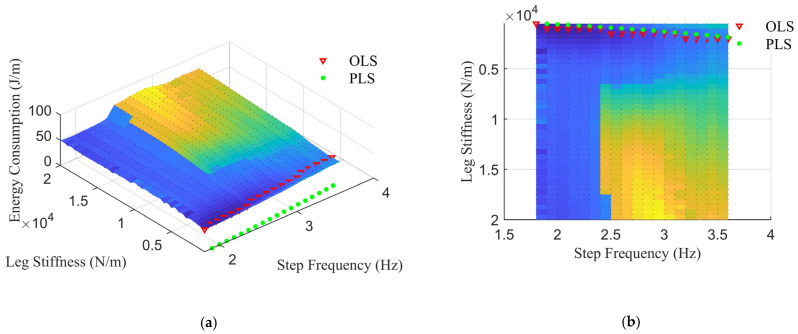
Energy consumption of robot locomotion with different gait frequency and leg stiffness. (**a**) Isometric view; (**b**) bottom view. The red inverted triangles denote the Optimal Leg Stiffness (OLS) for a certain gait frequency where the energy consumption is minimum; green points indicate the Preferred Leg Stiffness (PLS) obtained from the Equation (20).

To observe the influence of leg stiffness on legged locomotion performance, a quadrupedal robot simulation model was constructed based on Matlab^®^ and Simscape^®^. The mass and geometric dimensions were set according to the robot experimental setup. The robot walked with different step frequencies and leg stiffness using a trotting gait at a speed of 2 m/s. The energy consumption of the robot locomotion was normalized by the displacement on the ground surface. The preferred leg stiffness to obtain minimum energy consumption at a given gait frequency was investigated and compared with the calculated leg stiffness using the Equation (20). The simulation result in Figure 11 shows that the simulated optimal leg stiffness is consistent with the calculated results.

## 6. Conclusions

Compliant legged locomotion has recently become an emerging area of interest in the field of robotics. Few studies, however, have been carried out on the planning and implementation of leg compliance with the consideration of ground stiffness. As mentioned in the introduction section, the main challenge is the lack of rapid and affordable ground compliance estimation methods and of reasonable principles for leg compliance planning. In this paper, a systematic compliance planning and implementation method for the quadrupedal robot is proposed to plan and control the leg compliance continuously with the consideration of the ground through surface stiffness estimation. In this way, the compliant robot leg behaves naturally following bio-inspired principles, and the performance is improved in terms of locomotion efficiency and rhythmicity. The effectiveness of the proposed control method has been shown through simulations and experimental results on a hydraulic quadruped robot. The proposed method can also be extended to other legged robots actuated by hydraulic systems or motors where both the torque and compliance are controllable.

Future work will include the development of the proposed control architecture for a practical hydraulic quadruped robot, walking and running in a more challenging ground environment.

## Data Availability

This paper did not generate research data to share.
